# School readiness and its determinants among children 4 to 6 years in Zanzibar: a baseline population-based cross-sectional survey

**DOI:** 10.3389/fpubh.2026.1832373

**Published:** 2026-07-01

**Authors:** Edward Kija, Davis Amani, Mariam Ngaula, Elias Bukundi, Alexander Mwijage, Halima Tahir, Ester Elisaria, Hamad Bakari, Yahya Mselem, Ali Omar, Shehe Mohammed, Khamis Ally Abeid, Maryam Juma, Ibrahim Kabole, Mucho Mizinduko

**Affiliations:** 1 Department of Pediatrics and Child Health, Muhimbili University of Health and Allied Sciences (MUHAS), Dar Es Salaam, Tanzania; 2 Department of Epidemiology and Biostatistics, Muhimbili University of Health and Allied Sciences (MUHAS), Dar Es Salaam, Tanzania; 3 Institute of Traditional Medicine, Muhimbili University of Health and Allied Sciences (MUHAS), Dar Es Salaam, Tanzania; 4 Department of Health Systems, Impact Evaluation and Policy Analysis, Ifakara Health Institute, Dar Es Salaam, Tanzania; 5 Ministry of Education and Vocational Training, Revolutionary Government of Zanzibar, Zanzibar, Tanzania; 6 Ministry of Health, Revolutionary Government of Zanzibar, Zanzibar, Tanzania; 7 Ministry of Community Development, Gender, Elderly and Children, Revolutionary Government of Zanzibar, Zanzibar, Tanzania; 8 Department of Early Childhood, Primary, Inclusive and Special Needs Education, State University of Zanzibar, Zanzibar, Tanzania; 9 Department of Paediatrics and Child Health, State University of Zanzibar, Zanzibar, Tanzania; 10 Presidential Delivery Bureau, Revolutionary Government of Zanzibar, Zanzibar, Tanzania

**Keywords:** early childhood development, IDELA, nurturing care, school readiness, Zanzibar

## Abstract

Early childhood development (ECD) is essential for lifelong learning and wellbeing. School readiness a key component of ECD is influenced by the dynamic interaction among children, their families, communities, and early learning environments. The evidence on these determinants is limited in many low and middle-income countries. A cross-sectional household survey was conducted to establish a baseline assessment of school readiness among children aged 4–6 years in Zanzibar. Social demographic data was collected using a structured questionnaire adapted from the UNICEF Multiple Indicator Cluster Survey. School readiness was measured using the International Development and Early Learning Assessment (IDELA) with household vulnerability being measured by FHI360 Household Vulnerability Assessment Tool (HVAT). A total of 362 child-caregiver pairs were enrolled, with children having a median age of 60 months (IQR: 54–65) and caregivers a mean age of 34.8 years (SD: 8.5. About 47% of children were living in households with lowest degree of vulnerability. Overall, 23% of children were classified as struggling in school readiness with the poorest performance observed in emergent literacy (40%) and social–emotional development (37%). Children residing in rural areas and in more vulnerable households were significantly more likely to be in the struggling category compared with their urban and less vulnerable counterparts (*p* < 0.01). Multinomial logistic regression showed that increasing child age was positively associated with school readiness, with each additional month increasing the relative risk of being in the emerging (RRR=1.17, 95% CI: 1.11–1.22) and mastering groups (RRR = 1.25, 95% CI: 1.17–1.34) compared with struggling (*p* < 0.001). Children living in urban areas were substantially more likely to be in the emerging (RRR = 2.93, 95% CI: 1.56–5.50) and mastering groups (RRR = 4.56, 95% CI: 1.85–11.20) than rural children. Household vulnerability was associated with lower school readiness, with vulnerable children having a reduced likelihood of being in the emerging group relative to struggling (RRR = 0.51, 95% CI: 0.28–0.91). A substantial proportion of preschool children in Zanzibar enter formal schooling without adequate readiness. Strengthening ECD strategies is required, with priority given to rural communities and vulnerable households to advance progress toward universal access to quality ECD and pre-primary education.

## Introduction

Early childhood development (ECD) is widely recognized as a critical foundation for lifelong learning, health, wellbeing, and economic productivity (1). Among the core components of ECD is school readiness, defined as a child's holistic preparedness to successfully engage in formal education across multiple domains, including physical health and motor development, cognitive and general knowledge, language and communication skills, social and emotional competence, and approaches to learning (2–4). Importantly, school readiness is not determined solely by child-level characteristics but rather emerges from dynamic interactions among children, their families, communities, and early learning environments (4).

Globally, the importance of early learning is reinforced by Sustainable Development Goal (SDG) 4.2, which calls for universal access to quality early childhood development and pre-primary education by 2030 (5). Despite this commitment, progress remains uneven, particularly in sub-Saharan Africa, where preschool enrolment among children aged 3–5 years remains at approximately 30% (6). As a result, many children enter primary school without foundational literacy, numeracy, and socio-emotional skills, placing them at increased risk of poor educational trajectories. In Tanzania, national policy frameworks, including the Child Development Policy (2008) and subsequent ECD strategies, acknowledge persistent gaps in school readiness, particularly among children transitioning to Standard one, with an estimated 35–45% lacking basic competencies based on Early Childhood Development Index (ECDI) indicators (7, 8).

Tanzania has made notable progress in expanding access to early childhood education, with pre-primary enrolment increasing from 24.5% in 2004 to approximately 77% in recent years (9). In Zanzibar, the abolition of school fees in 2015 and 2018 contributed to substantial increases in enrolment in both public and private schools between 2017 and 2021 (10). However, learning outcomes remain concerning: nationally, only 12% of Tanzanian second graders could read with comprehension, while only 6.5% met the oral reading fluency, 1.3% met the no-wording reading, and 8% passed the addition and subtraction benchmarks (11). These findings suggest that increased access has not been matched by adequate school readiness at entry, underscoring the need to examine developmental foundations prior to formal schooling.

Evidence shows that school readiness is shaped by interacting child, household, and environmental-level factors (12). At the child level; age, sex, health and nutritional status particularly stunting is strongly associated with cognitive, language, and socio-emotional development, with stunted children demonstrating poorer school readiness outcomes (13, 14). At the household level, maternal education and socioeconomic status influence children's early learning through access to nutrition, healthcare, and stimulating home environments, with studies in Tanzania showing children from higher socioeconomic households significantly outperform disadvantaged peers in early reading and mathematics (15, 16). At the environment level, access to and quality of pre-primary education, geographic location, and community-level health and social services further shape developmental trajectories (17). In low- and middle-income countries, these disparities are often amplified by structural inequalities, with children from rural areas and socioeconomically disadvantaged households facing greater constraints to achieving their developmental potential (12, 18). In a study conducted in Brazil among children with a mean age of 69 months, more than half were classified in the emergent category, with most insufficiency observed in the executive function category (19). Despite growing investments in ECD, unmet needs remain substantial, and context-specific evidence to guide effective, equitable interventions is limited.

In Zanzibar, children constitute a large and diverse segment of the population across the islands of Unguja and Pemba. While multiple ECD initiatives have been introduced, such as pre-primary education, maternal and child health programs, there is limited systematic evidence on current levels of school readiness among children aged 4–6 years and the factors associated with developmental outcomes at this critical transition point. This study, therefore, aimed to provide a baseline assessment of school readiness and its associated child-, household-, and environment-level factors among children aged 4–6 years in Zanzibar, ahead of planned ECD policy restructuring. The revolutionary government of Zanzibar is planning to improve on its policies to ensure appropriate interventions for optimizing ECD are designed and implemented. The plan has started with the conduction of this baseline study. The findings will inform the Revolutionary Government of Zanzibar and other stakeholders by identifying priority gaps, guiding the design of targeted and contextually appropriate interventions, and supporting the development of evidence-informed ECD policies and programs.

## Methods

### Design and setting

This was a cross-sectional household survey conducted in Zanzibar between September and October 2024. Zanzibar has a total population of 1,889,773 among whom, 963,498 live in rural areas and 926,275 live in urban areas (20). The 2022 Population and Housing Census established that there were 380,260 households with about half (193,633 households) in rural areas and the remaining half in urban areas. The average household size was determined to be five. Children aged 0–8 years constituted at least a quarter of the population (26%). Administratively, Zanzibar has five regions and eleven districts across two main islands of Unguja and Pemba. The eleven districts are further subdivided into 388 enumeration areas (Shehias).

### Study population

The target population for the survey was child-parents/caregivers' pairs of children aged 4–6 years residing in Zanzibar. Children aged 4–6 is the best timing to assess school readiness as this is the age children are expected to be in pre-school programs The pair was eligible if they had an address in Zanzibar and had lived in the area for at least 1 year.

### Sample size and sampling techniques

The sample size was determined to be 360 using the proportion of children in grade two in Zanzibar who could read with comprehension (21) and a design effect of 2 to account for the design of the study.

We deployed a two-stage stratified cluster sampling technique to sample households across the 388 shehias. In the first stage, a total of 24 enumeration areas (Shehias) were randomly selected from the 11 districts. The selection took into account probability proportional to size of the Shehia and rural/urban mix. Of the 24 Shehias, 14 Shehias were from the rural areas and 10 from the urban areas.

In the second stage, a systematic sampling technique was used to select a fixed number of households in each enumeration area (Shehia). In this method, every *K*^*th*^ household (where K–systematic sampling interval, equals to total number of households in a Shehia over sample size of 15 households per Shehia) was selected in a predetermined direction starting from a conveniently selected point (such as a mosque, a government office, a huge tree etc.) in the cluster until 15 households with eligible participants have been identified. In each of the households sampled, one child was selected for the interview. If the household had more than one eligible child, a simple random sampling technique was used to select a participant for interviews. If there was no one in the house at the time of interview, the next nearby household was selected. If all the nearby households had no person at the time of interview, the K^th^ factor was applied to select the next household.

### Data collection

We recruited 362 child-parent/caregiver pairs for children aged 4–6 years with their birth dates verified using child's health cards and word of primary caregiver. Data collection was conducted by trained research assistants using open data kit (ODK) mounted tablets. Prior to the commencement of data collection, all research assistants attended a 6-day training course that focused on study objectives, recruitment procedures, survey methods, and ethical considerations.

Data collection was done using a structured questionnaire through face-to-face interviews with participants/caregivers. The questionnaire constituted a set of questions on child's and household's demographics mostly adopted from the UNICEF Multi indicator cluster survey questionnaire (22). We collected data on the age of the child, age of the parent/caregiver, sex of the child, sex of the parent/caregiver, marital status of the caregiver, education level of the caregiver, occupation of the caregiver, residence, and the main source of income of the household. We also adopted other various tools to measure different parameters to assess children school readiness and factors that may be associated with readiness. The International Development and Early Learning Assessment (IDELA) tool, developed by Save the Children, was used to determine the school readiness of the child (23). The IDELA tool assesses child's school readiness and competencies in five key areas: motor, language and early literacy, math and problem solving, socio-emotional development, and approaches to learning. These competencies are assessed objectively by interviewing and observing a child performing a series of tasks for each domain (23, 24). Using categories suggested in the IDELA tool, we categorized children scoring 0 to 24% as struggling (Child shows limited skills in the domain), 25–74% as emerging (Child demonstrates some skills but not fully confident), and 75–100% as mastering (Child demonstrates strong skills) (23). To measure household vulnerability, we used the FHI360 Household Vulnerability Assessment Tool (HVAT) (25). The HVAT would classify a child whose household scores less than 25% in vulnerability assessment as non-vulnerable and can graduate, those whose households score between 25% and 49% as slightly vulnerable, those whose households score 50% to 74% as moderately vulnerable, and those scoring 75% to 100% as critically vulnerable (25).

### Data analysis

Descriptive statistics reporting on proportions and measures of central tendency as deemed appropriate were used to summarize the data. Pearson's Chi-squared test was used to establish preliminary association between different factors and child's school readiness score. Multinomial logistic regression analysis was used to determine factors associated with school readiness, with IDELA score categories as the outcome of interest. Although the outcome variable was ordinal categorical, a multinomial logistic regression model was preferred over an ordinal logistic regression model because the proportional odds assumption was violated. The final regression model constituted the following variables: age of the child in months, sex of the child, place of residence of the child, and household vulnerability level. Variables were selected before developing the model based on conceptualization. Education, occupation, and household vulnerability level were not included simultaneously in the multivariable model. The vulnerability score is a composite index that incorporates, in part, education and occupation, resulting in high collinearity with these variables. Including both, the composite score and its constituent components in the same model, would introduce multicollinearity, leading to unstable estimates and inflated standard errors, as well as statistical redundancy that compromises model parsimony. Predictors with a *p* value greater than 0.20 in the bivariate analyses were excluded from the model. These included knowledge on protection of children against illnesses, caregivers' affection and responsive stimulation, responsive health and nutrition, and practicing appropriate disciplinary measures. All statistical analyses were conducted using Stata Statistical Software: Release 15 (StataCorp, 2017; College Station, TX). All statistical tests were two-sided, and a *p*-value of < 0.05 was considered statistically significant.

The study was approved by the Muhimbili University of Health and Allied Sciences Research and Ethics committee, MUHAS-REC-09-2024-2451, Office of the second Vice President of the Revolutionary Government of Zanzibar CCA.33/411/01/56 and Zanzibar Health Research Institute Ref: NO. ZAHREC/01/PR/OCT/2024/34.

## Results

### Socio-demographic characteristics of the study participants

We enrolled 362 child-caregiver pairs with children having a median (IQR) age of 60.0 (54.0–65.0) months, and their caregivers had a mean (SD) age of 34.8 (8.5) years. Nearly nine of every ten caregivers (87%) who responded to the questionnaire were females, and four out of every ten caregivers (43%) had primary or no formal education. About half of the households had learning materials, ate all three food groups, and had three or more meals on a typical day. At most, half (47%) of the children lived in households with the lowest degree of vulnerability ( ≤ 25%) determined by using vulnerabilities in economic strength, food security and nutrition, health, shelter, education of the head of household, psychosocial support, basic care, child protection, and legal support (Table 1).

**Table 1:** General Characteristics of the study participants.

Characteristic	Overall, *N* = 362^1^	Pemba, *N* = 120^1^	Unguja, *N* = 242^1^
**Age of child in months–median (IQR)**	60.0 (54.0, 65.0)	63.0 (57.8, 70.0)	60.0 (54.0, 64.8)
**Age of a parent in years–mean (sd)**	34.8 (8.5)	33.8 (7.7)	35.3 (8.9)
Sex of a child			
Female	182 (50)	65 (54)	117 (48)
Male	180 (50)	55 (46)	125 (52)
Sex of a caregiver			
Female	347 (96)	115 (96)	232 (96)
Male	15 (4.1)	5 (4.2)	10 (4.1)
Marital status of a caregiver			
Married	314 (87)	107 (89)	207 (86)
Single/divorced/widow	48 (13)	13 (11)	35 (14)
Education level of caregiver			
No formal education	46 (13)	17 (14)	29 (12)
Primary	110 (30)	45 (38)	65 (27)
Secondary/Higher	206 (57)	58 (48)	148 (61)
Occupation of a caregiver			
Agriculture	49 (14)	26 (22)	23 (9.5)
Employed	20 (5.5)	4 (3.3)	16 (6.6)
Housewife/husband	117 (32)	38 (32)	79 (33)
Self–employed/Business	176 (49)	52 (43)	124 (51)
Residence			
Rural	214 (59)	95 (79)	119 (49)
Urban	148 (41)	25 (21)	123 (51)
Main source of household income			
Formal employment/business/commercial farming	24 (6.6)	10 (8.3)	14 (5.8)
Petty Business	55 (15)	26 (22)	29 (12)
Informal employment/peasantry/casual laborer	69 (19)	24 (20)	45 (19)
Remittances (Pension, Gratuity, Donations)	59 (16)	35 (29)	24 (9.9)
None	155 (43)	25 (21)	130 (54)
Household has child's learning materials			
Yes	170 (47)	38 (32)	132 (55)
No	192 (53)	82 (68)	110 (45)
Type of foods routinely consumed by the family			
All food groups	169 (47)	38 (32)	131 (54)
Two food groups	156 (43)	57 (48)	99 (41)
One food group	35 (9.7)	24 (20)	11 (4.6)
How many meals does the household have in a day?			
Three or more meals	188 (52)	47 (39)	141 (59)
Two meals per day	152 (42)	63 (53)	89 (37)
One meal	19 (5.3)	9 (7.6)	10 (4.1)
Some days no meal	1 (0.3)	0 (0)	1 (0.4)
Household vulnerability score			
0–25% (non – vulnerable)	170 (47)	43 (36)	127 (52)
26–75% (vulnerable)	192 (53)	77 (64)	115 (48)

1 Mean (SD); Median (IQR); n (%).

### School readiness of children aged 4 – 6 years

Using the IDELA tool, the survey conducted a comprehensive evaluation of school readiness among children aged 4–6 years. This tool is designed to measure various developmental areas critical for children's preparedness to enter school, ensuring that their skills and abilities are appropriately assessed. Of the 362 children assessed 23% were in the struggling category with the most affected domains being social-emotional (37% in the struggling category) and emergent literacy (40% in the struggling category). Scores varied across different structural and socio-demographic strata. Compared to rural communities, children residing in urban communities were more likely to be in the mastering group (13% in the urban vs 8.9% in the rural group) and in the emerging group (73% in the urban vs 62% in the rural). Furthermore, children in the rural communities were more likely to be in the struggling group than children in the urban communities (29% in the rural vs. 14% in the urban) and these differences were statistically significant (p = 0.003). A similar trend was seen across individual domains, where children in urban communities were more likely to be in the mastering and emerging groups than children in rural communities (Table 2).

**Table 2:** School readiness scores by domains disaggregated by residence in rural or urban communities.

Characteristic	Overall, *N* = 362^1^	Rural, *N* = 214^1^	Urban, *N* = 148^1^	*p*–value^2^
Final score				
Struggling	84 (23)	63 (29)	21 (14)	0.003
Emerging	240 (66)	132 (62)	108 (73)	
Mastering	38 (10)	19 (8.9)	19 (13)	
Socioemotional				
Struggling	134 (37)	94 (44)	40 (27)	0.005
Emerging	194 (54)	102 (48)	92 (62)	
Mastering	34 (9.4)	18 (8.4)	16 (11)	
Emergent literacy				
Struggling	143 (40)	99 (46)	44 (30)	0.002
Emerging	179 (49)	98 (46)	81 (55)	
Mastering	40 (11)	17 (7.9)	23 (16)	
Emergent numeracy				
Struggling	97 (27)	70 (33)	27 (18)	< 0.001
Emerging	204 (56)	119 (56)	85 (57)	
Mastering	61 (17)	25 (12)	36 (24)	
Fine motor				
Struggling	54 (15)	40 (19)	14 (9.5)	0.009
Emerging	186 (51)	113 (53)	73 (49)	
Mastering	122 (34)	61 (29)	61 (41)	

^1^n (%) ^2^Pearson's Chi–squared test.

Variations of school readiness across the two islands of Zanzibar (Pemba and Unguja) were also assessed, where no significant differences between children were found. However, Unguja had a higher proportion of children in the emerging category than in Pemba (69% vs 62%), whereas Pemba had slightly more children in the mastering category than Unguja (14% vs. 8.7%). Scores varied across the four domains with good performance on the fine motor domain (34% in the mastering group, 51% in emerging group, and 15% in struggling group) and poor performance on the socio-emotional domain (9.4% in the mastering group, 54% in the emerging group, and 37% in the struggling group) (Table 3).

**Table 3:** School readiness scores by domains disaggregated by residence in the islands (Pemba vs Unguja).

Characteristic	Overall, *N* = 362^1^	Pemba, *N* = 120^1^	Unguja, *N* = 242^1^	*p*–value^2^
Final score				
Struggling	84 (23)	29 (24)	55 (23)	0.2
Emerging	240 (66)	74 (62)	166 (69)	
Mastering	38 (10)	17 (14)	21 (8.7)	
Socio emotional				
Struggling	134 (37)	50 (42)	84 (35)	0.2
Emerging	194 (54)	56 (47)	138 (57)	
Mastering	34 (9.4)	14 (12)	20 (8.3)	
Emergent literacy				
Struggling	143 (40)	46 (38)	97 (40)	>0.9
Emerging	179 (49)	61 (51)	118 (49)	
Mastering	40 (11)	13 (11)	27 (11)	
Emergent numeracy				
Struggling	97 (27)	26 (22)	71 (29)	0.12
Emerging	204 (56)	68 (57)	136 (56)	
Mastering	61 (17)	26 (22)	35 (14)	
Fine motor				
Struggling	54 (15)	21 (18)	33 (14)	0.06
Emerging	186 (51)	51 (43)	135 (56)	
Mastering	122 (34)	48 (40)	74 (31)	

^1^n (%) ^2^Pearson's Chi–squared test.

Using household vulnerability score, we compared children school readiness score across the two levels of vulnerability. Overall, children in more vulnerable households were more likely to be in the struggling group compared to children in the non-vulnerable households (29% of children in more vulnerable households were in the struggling group vs 16% of children in less vulnerable households) and this difference was statistically significant (p = 0.008). A similar finding was observed across the four domains of school readiness (Table 4).

**Table 4:** School readiness scores by domains disaggregated by household vulnerability score.

Characteristic	Overall, *N* = 362^1^	Non–vulnerable households, *N* = 170^1^	Vulnerable households, *N* = 192^1^	*p*–value^2^
Final score				
Struggling	84 (23)	28 (16)	56 (29)	0.008
Emerging	240 (66)	126 (74)	114 (59)	
Mastering	38 (10)	16 (9.4)	22 (11)	
Socio emotional				
Struggling	134 (37)	60 (35)	74 (39)	0.7
Emerging	194 (54)	92 (54)	102 (53)	
Mastering	34 (9.4)	18 (11)	16 (8.3)	
Emergent literacy				
Struggling	143 (40)	64 (38)	79 (41)	0.7
Emerging	179 (49)	88 (52)	91 (47)	
Mastering	40 (11)	18 (11)	22 (11)	
Emergent numeracy				
Struggling	97 (27)	37 (22)	60 (31)	0.11
Emerging	204 (56)	104 (61)	100 (52)	
Mastering	61 (17)	29 (17)	32 (17)	
Fine motor				
Struggling	54 (15)	17 (10)	37 (19)	0.04
Emerging	186 (51)	90 (53)	96 (50)	
Mastering	122 (34)	63 (37)	59 (31)	

^1^n (%) ^2^Pearson's Chi–squared test.

### Factors associated with school readiness of children aged 4–6 years

Multinomial logistic regression analysis was used to determine the factors associated with school readiness among children aged 4–6 years. The final regression model constituted the following variables: age of the child in months, sex of the child, residence of the child, and household vulnerability level. Due to collinearity, other variables were not included in the final model.

The age of the child in months was associated with school readiness as assessed by IDELA scores. For a month increase in age, there was a 17% higher relative risk of being in the emerging group as compared to being in the struggling group (RRR = 1.17, 95%CI: 1.11–1.22; p < 0.001) whereas for the same one-month increase in age, there was 25% higher relative risk of being in the mastering group as compared to being in the struggling group (RRR = 1.25, 95%CI: 1.17–1.34; p < 0.001). The sex of the child was also associated with school readiness: male children had on average, 32% lower relative risk of being in the emerging group as compared to being in the struggling group when compared to female children (RRR = 0.68, 95%CI: 0.39, 1.19; p-value = 0.18). Also, male children had on average 55% lower relative risk of being in the mastering group as compared to being in the struggling group when compared to female children (RRR = 0.45, 95%CI: 0.19–1.05; p-value = 0.07). However, the association between the sex of the child and IDELA scores did not attain the set statistical significance.

Residence, as to whether the child lives in a rural or urban residence, was also determined to be a factor associated with school readiness. Children residing in urban communities were three times as likely to be in the emerging group as compared to being in the struggling group when compared to children residing in rural communities (RRR = 2.93, 95%CI: 1.56–5.50; p < 0.001). Also, children in the urban communities were four and a half times as likely to be in the mastering group as compared to being in the struggling group when compared with children in the rural communities (RRR = 4.56, 95%CI: 1.85–11.20; p < 0.001). Furthermore, children residing in vulnerable households had 49% lower relative risk of being in the emerging group than in the struggling group when compared with children residing in non-vulnerable households (RRR = 0.51, 95%CI: 0.28–0.91; p = 0.023). Also, comparing the likelihood of being in the mastering group versus being in the struggling group, children from vulnerable households had 29% lower relative risk of being in the mastering group than in the struggling group when compared with children from non-vulnerable households. However, this association did not attain a statistical significance (RRR = 0.71, 95%CI: 0.29–1.69; p = 0.43) (Table 5).

**Table 5:** Factors associated with school readiness among children aged 4–6 years.

	Emerging vs struggling		Mastering vs struggling	
Characteristic	RRR^1^ (95% CI^2^)	*p*–value	RRR^1^ (95% CI^2^)	*p*–value
Age of child (months)	1.17 (1.11, 1.22)	< 0.001	1.25 (1.17, 1.34)	< 0.001
Sex of a child				
Female				
Male	0.68 (0.39, 1.19)	0.18	0.45 (0.19, 1.05)	0.07
Residence				
Rural				
Urban	2.93 (1.56, 5.50)	< 0.001	4.56 (1.85, 11.2)	< 0.001
Vulnerability index				
Less vulnerable				
More vulnerable	0.51 (0.28, 0.91)	0.023	0.71 (0.29, 1.69)	0.43

^1^Relative Risk Ratio, ^2^CI, Confidence Interval.

## Discussion

This study assessed school readiness among preschool children in Zanzibar using the International Development and Early Learning Assessment (IDELA) tool and found that nearly one in four children (23%) were classified as struggling, with the poorest performance observed in emergent literacy and social-emotional development domains. School readiness was associated with age, place of residence (urban vs rural), and household vulnerability. Older children were significantly more likely to be classified as emerging or mastering compared with struggling, while children residing in rural areas and those from vulnerable households had substantially lower readiness levels. Urban residence was consistently associated with higher readiness across categories, and no meaningful differences were observed between the two islands of Unguja and Pemba.

The IDELA tool has been widely used to measure school readiness among preschool children. Studies in similar settings showed comparable findings in terms of the proportion of children in the three IDELA categories, reflecting similar limitations in early childhood education systems across the region. A report on school readiness assessment using IDELA tool in Uganda found about one in ten children (12%) was in a mastering group which is similar to the findings of this survey. However, the report in Uganda found a comparatively smaller proportion of children in the struggling group (5%) than observed in this survey (26). Of note, while this survey included children as young as 4 years, the assessment in Uganda included older children (5.6 years to 6.5 years). This may signal that school readiness is associated with the older age of the child.

Using IDELA scores, the survey has shown that there is a positive gradient in school readiness as the child gets older. This finding aligns with developmental theory and empirical evidence from LMICs, which consistently show maturation-related gains in cognitive, language, and socio-emotional skills (16, 27, 28). Sex of the child may determine IDELA score in different contexts due to cultural differences. However, studies have not shown such differences in overall IDELA scores with marginal differences in individual domain scores (29, 30). The current survey has shown similar trends with a higher likelihood of being in the higher category of scores as the child gets older but with girls scoring slightly higher than boys using the overall IDELA score. These findings suggest that interventions to enhance school readiness should take into account the inclusivity of age and gender among children. Including younger children in these interventions may yield greater benefits.

Also, residence emerged as a predictor of school readiness, with urban children substantially more likely to be in emerging or mastering categories compared with their rural counterparts. This finding mirrors results from studies in Kenya, Uganda, Rwanda, and global pooled analyses, which attribute urban advantages to better access to early childhood education services, learning materials, trained caregivers, and stimulating home environments (1, 31, 32). Programs to improve school readiness in rural communities should be instituted in efforts to improve their school performance. Such programs have been proven to be effective in settings like Zanzibar. A school readiness program piloted in mainland Tanzania observed higher scores among children who were put in intervention communities than children who received the existing standard schooling or received nothing. This program incorporated a community play-based learning to enhance foundation skills on literacy, cognitive development and social emotional readiness to prepare children for formal schooling (30). A similar program in Rwanda which combined standard child classroom tools and focus group discussions for parents proved to be beneficial in enhancing school readiness for children (29).

Household vulnerability has also been determined to impact school readiness of children in some previous studies (29, 33). A publication by the Organization for Economic Co-operation and Development in 2019 indicated that children from poor households lack access to learning materials and are less likely to interact with adults for activities that will stimulate learning (34). Similarly, the current survey found that children from vulnerable households were less likely to be ready for school compared to children from non-vulnerable households. The vulnerable households are more likely to have poor nutrition and inadequate access to learning materials which in turn may impact school readiness. Thus, to realize children's full potential, it is important for social welfare programs to institute measures to reduce household vulnerability (35–37).

Due to various potential limitations, findings from this study should be interpreted with caution. The cross-sectional design limits causal inference, and observed associations should be interpreted as correlational. Additionally, IDELA assessments capture performance at a single point in time and may not fully reflect developmental trajectories. Future research should prioritize longitudinal designs to examine developmental trajectories and causal pathways linking household, community, and programmatic factors to school readiness. Evaluations of intervention effectiveness, particularly in rural and high-vulnerability settings, are also needed. Further qualitative research could deepen understanding of caregiver practices, cultural norms, and systemic barriers influencing early learning in Zanzibar. Integrating measures of preschool quality and home stimulation into future assessments would provide a more comprehensive picture of determinants of school readiness.

However, this study had several strengths to put into consideration. The first strength of this study is the use of validated measures such as the IDELA and HVAT tools which allow for comparability across settings and was interviewer administered not caregiver reported. Secondly, the use of multinomial logistic regression enabled a nuanced examination of factors associated with different levels of readiness rather than relying on binary classifications. Third, the relatively large sample size, robust sampling approach, and inclusion of children from both Unguja and Pemba islands enhance the internal validity and generalizability of the findings across Zanzibar.

## Conclusion and Recommendations

This study has shown that a considerable proportion of preschool children in Zanzibar enter formal schooling without adequate readiness, with pronounced deficits in emergent literacy and social-emotional development. Observed inequities are linked to age, place of residence, and household vulnerability. These findings provide strong, context-specific evidence to support urgent strengthening of early childhood development strategies in Zanzibar. Priority should be given to younger children in rural communities, and vulnerable households through integrated, community-based early learning and nurturing care interventions. Embedding these interventions within existing social, health, and education platforms will support progress toward universal access to quality early childhood development and pre-primary education by 2030.

## Data Availability

The raw data supporting the conclusions of this article will be made available by the authors, upon request.
